# Expression and prognostic impact of matrix metalloproteinase-2 (MMP-2) in astrocytomas

**DOI:** 10.1371/journal.pone.0172234

**Published:** 2017-02-24

**Authors:** Rahimsan K. Ramachandran, Mia D. Sørensen, Charlotte Aaberg-Jessen, Simon K. Hermansen, Bjarne W. Kristensen

**Affiliations:** 1 Department of Pathology, Odense University Hospital, Odense, Denmark; 2 Department of Clinical Research, University of Southern Denmark, Odense, Denmark; Swedish Neuroscience Institute, UNITED STATES

## Abstract

Astrocytomas are the most frequent primary brain tumors in adults, and despite aggressive treatment patients often experience recurrence. Survival decreases with increasing tumor grade, and especially patients with grade IV glioblastoma have poor prognosis due to the aggressive character of this tumor. Matrix metalloproteinase-2 (MMP-2) is an extracellular matrix degrading enzyme which has been shown to play important roles in different cancers. The aim of this study was to investigate the expression and prognostic potential of MMP-2 in astrocytomas. Tissue samples from 89 patients diagnosed with diffuse astrocytoma, anaplastic astrocytoma and glioblastoma were stained immunohistochemically using a monoclonal MMP-2 antibody. The MMP-2 intensity in cytoplasm/membrane was quantified by a trained software-based classifier using systematic random sampling in 10% of the tumor area. We found MMP-2 expression in tumor cells and blood vessels. Measurements of MMP-2 intensity increased with tumor grade, and MMP-2 expression was found to be significantly higher in glioblastomas compared to normal brain tissue (p<0.001), diffuse astrocytomas (p<0.001) and anaplastic astrocytomas (p<0.05). MMP-2 expression was associated with shorter overall survival in patients with grade II-IV astrocytic tumors (HR 1.60; 95% CI 1.03–2.48; p = 0.036). In glioblastoma, high MMP-2 was associated with poorer prognosis in patients who survived longer than 8.5 months independent of age and gender (HR 2.27; 95% CI 1.07–4.81; p = 0.033). We found a positive correlation between MMP-2 and tissue inhibitor of metalloproteinases-1 (TIMP-1), and combined MMP-2 and TIMP-1 had stronger prognostic value than MMP-2 alone also when adjusting for age and gender (HR 2.78; 95% CI 1.30–5.92; p = 0.008). These findings were validated in bioinformatics databases. In conclusion, this study indicates that MMP-2 is associated with aggressiveness in astrocytomas and may hold an unfavorable prognostic value in patients with glioblastoma.

## Introduction

World Health Organization (WHO) grade III and IV gliomas are primary brain tumors with the highest incidence in adults [1]. Among these high-grade gliomas, the grade IV astrocytic tumor known as glioblastoma multiforme (GBM) is the most common, aggressive, and deadly tumor. The therapeutic strategy for GBM involves surgery, irradiation, and chemotherapy [2], and this treatment regime has resulted in an increase in median progression-free and overall survival [3, 4]. In GBMs, methylation of the O6-methylguanine-DNA-methyltransferase (MGMT) promoter is used to decide whether elderly patients will benefit from treatment with temozolomide instead of radiotherapy [5–7]. Yet, valuable biomarkers lack when it comes to the whole population of GBM patients. The search for new and better prognostic and predictive biomarkers is therefore ongoing.

The key enzymes involved in degradation of extracellular matrix (ECM) are the matrix metalloproteinases (MMPs) which in a complex interplay with their inhibitors, tissue inhibitors of metalloproteinases (TIMPs), play a major role in both normal physiological functions and cancer-related processes such as cell migration, inflammation, invasion, metastasis, angiogenesis, and proliferation [8, 9]. We [10] and others [11, 12] have previously reported that low levels of TIMP-1 are beneficial for patients with astrocytic brain tumors. Similarly, the majority of the over 20 members of the MMP family [8, 13] including MMP-2 have been associated with tumor progression/aggressiveness in several cancers such as gastric [14], esophageal [15], breast [16], prostate [17], lung [18], bladder [19], and ovarian carcinoma [20]. MMP-2 cleaves different gelatins, collagens, and other matrix substrates important for the integrity of basement membrane and ECM [21, 22]. In glioma, MMP-2 is believed to be widely involved in tumor spreading, and previous studies have shown a correlation between MMP-2 and gliomas [23–26]. However, only nine to 35 GBM cases were included in these studies, and survival analysis was either not performed or was not investigated in separate WHO grades. Further, observer-based scoring was often used as the quantitative method. Further knowledge is therefore needed to decipher the influence of MMPs and TIMPs on tumorigenesis and progression in gliomas especially GBMs.

The aim of this study was to investigate the prognostic influence of MMP-2 by performing automated quantitative immunohistochemistry on tumor samples from 89 patients diagnosed with WHO grade II-IV astrocytic brain tumors. We have previously used advanced image analysis successfully [27–32], and to our knowledge, we are the first to quantify the expression levels of MMP-2 using this approach. MMP-2 expression was found to significantly increase with malignancy grade, and high levels correlated with poor prognosis in patients with grade II-IV astrocytomas. In patients with GBM, high levels of MMP-2 were significantly associated with poorer prognosis only in patients who survived longer than 8.5 months. High MMP-2 levels were correlated with high TIMP-1 score in GBMs; and the two proteins combined into a sum score seemed to be a stronger predictor of shorter overall survival compared to MMP-2 alone.

## Materials and methods

### Patients

Tissue samples were obtained from an astrocytoma cohort used and described in previous biomarker studies [10, 33, 34]. In total, 89 patients diagnosed with primary astrocytoma were included in the current study. All patients underwent initial surgery between 1995 and 2005 at the Department of Neurosurgery, Odense University Hospital, Denmark. Tumor samples were classified by two neuropathologist according to the WHO Classification from 2016 [1]. Of the 89 patients, 11 were diagnosed with diffuse astrocytoma (DA, WHO grade II), 6 with anaplastic astrocytoma (AA, WHO grade III) and 72 with GBM. Clinical data including gender, age, and survival was obtained from medical records. Astrocytomas where the available tumor tissue had been removed by ultrasonic aspiration or had been frozen prior to paraffin-embedment were excluded from the study to avoid tissue with degenerative features [35] and inferior morphology [36].

### Patient tissue

Tissue samples were fixed in 4% neutral-buffered formaldehyde and embedded in paraffin. Using a microtome, three μm sections were cut and routinely stained with hematoxylin-eosin to define the representative tumor regions.

Use of tissue was not prohibited by any patient according to the Danish Tissue Application Register. The study was approved by the Regional Committee on Health Research Ethics for Southern Denmark (Project-ID: S2DO9Oo8O) as well as the Danish Data Protection Agency (file number: 2009-41-3070), and it was performed in accordance with the Declaration of Helsinki.

### Immunohistochemistry

Three μm sections were stained on a Dako Autostainer Universal Staining System (Dako, Glostrup, Denmark). The sections were deparaffinized, and endogenous peroxidase activity was blocked in 1.5% hydrogen peroxide followed by heat-induced epitope retrieval in T-EG buffer for 15 minutes. The sections were then incubated overnight at 4°C with a MMP-2 antibody with affinity towards both latent MMP-2 (72 kDa) and active MMP-2 (66 kDa) (cloneVB3, Merck Millipore, Germany, diluted at 1:300). A polymeric horseradish peroxidase-conjugated system (PowerVision, Novocastra, United Kingdom) was used for detection of antigen-antibody complex followed by visualization with diaminobenzidine (DAB) as chromogen. Mayer’s haematoxylin was used for the nuclear counterstain, and coverslips were mounted with Aquatex. As a negative control, the primary antibody was omitted, showing no staining. Human ovarian carcinoma and bladder tissue were used as positive controls [18, 19].

### TIMP-1 score

Sections from patients with GBM (n = 72) were stained with the VT7 TIMP-1 monoclonal antibody (1:4000) in an earlier related study followed by observer-based scoring [10].

### Isocitrate dehydrogenase 1 status

Sections from patients with of DA, AA and GBM were stained with a mIDH1R132H antibody (mIDH1R132H, clone H14, Dionova, 1:100) to detect the most common isocitrate dehydrogenase (IDH) mutation using the BenchMark Ultra IHC/ISH staining system (Ventana Medical Systems, Inc, USA) as previously described [37, 38].

### Fixation experiment

To examine the influence of fixation time on the MMP-2 immunostaining, tissue samples from a patient with GBM were fixed in 4% neutral-buffered formaldehyde for 1, 6, 12, 24, or 48 h. No differences were observed in the MMP-2 intensity of the immunohistochemical reaction (S1 Fig).

### Image acquisition and analysis

All 89 slides were scanned on a Hamamatsu NanoZoomer 2.0–HT whole slide scanner (Hamamatsu Photonics K.K., Japan) at 20x magnification. Digital image analysis and quantitation were performed using Visiomorph software (Visiopharm, Denmark). Regions of interests containing tumor tissue were manually outlined followed by a systematic uniform random meander sampling using a sampling fraction of 10% and a 20x objective. The optimal sampling fraction was determined in a pilot study on 10 GBM specimens as previously described [30, 31]. Sample images were reviewed and excluded according to the following criteria: viable tumor tissue < 50%, staining artefacts > 50%, necrotic foci > 50%, and normal brain tissue > 50%.

**Fig 1:**
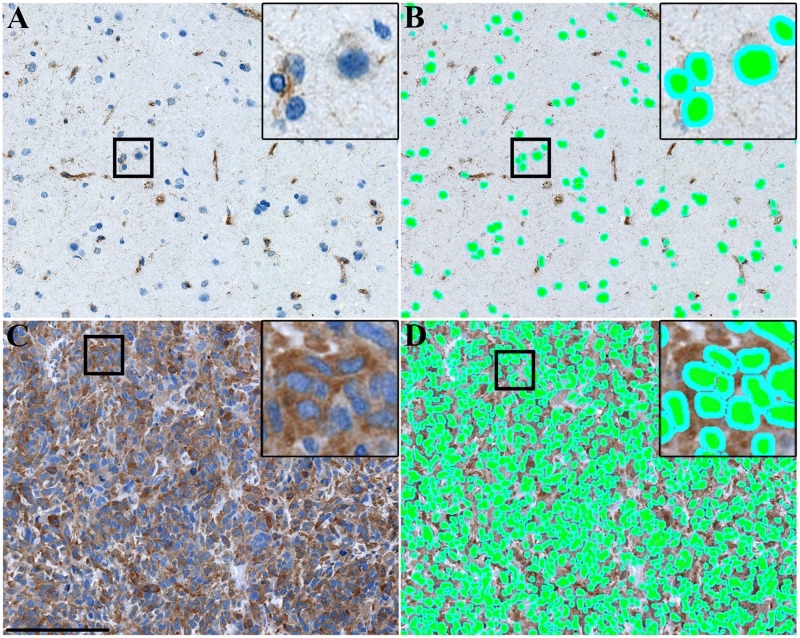
MMP-2 quantification using advanced software-based digital image analysis. Image analysis was performed using a pixel classifier based on Bayesian classification. The classifier was trained to identify nuclei (green color) and measure MMP-2 intensity in a three μm perimeter around the nuclei (turquoise color). **(A)** In normal brain tissue, mainly cells located in and/or around blood vessels showed MMP-2 immunopositivity. **(B)** The software classifier easily identified all cells for measurement of mean MMP-2 intensity. **(C)** In some GBMs, most tumor cells had intense MMP-2 staining. **(D)** Even in densely stained tumors, the software classifier successfully identified all cells for quantification of mean MMP-2 intensity. Scale bar 150 μm.

MMP-2 was quantified using a trained pixel classifier based on Bayesian classification. The mean MMP-2 intensity was measured in a three μm perimeter around the detected nuclei covering the cytoplasm/membrane or parts of the cytoplasm/membrane (Fig 1). The output data were given in RGB color model values ranging between 0–255 with low values equaling darker staining and high values equaling weaker staining. The average MMP-2 expression was calculated for all 89 tumors. Next, data was normalized for each patient by subtracting the measured RGB value from 255. The normalized values were then used in subsequent analyses. Patient data are available in S1 File.

### Patient dataset analysis

mRNA expressions of MMP-2 and TIMP-1 in grade II-IV primary astrocytomas and in non-tumor tissue were explored using GlioVis (https://gliovis.bioinfo.cnio.es). From the Cancer Genome Atlas (TCGA), data was available for 327 patients for analyzing the association between MMP-2 and malignancy grade [39]. For analysis of MMP-2 levels related to IDH status in grade II-IV astrocytomas, data was accessible for 346 patients [39]. For investigation of the association between MMP-2 and GBM subtype, mRNA expression data was available for 497 patients. Information on MGMT methylation status was available for 326 patients. For survival analyses in GBMs, datasets for MMP-2, TIMP-1, and c-MET (MET) were available for 497 patients [40]. Datasets were exported directly from GlioVis. Survival analyses were carried out using the median as cutoff value. Datasets used in the present study are available in S2 File.

### Cell culture

One GBM cell culture (T86), established in our laboratory [41–43], was used in the present study. The cell culture has been characterized by karyotyping and has the ability to generate new spheroids at clonal density and to differentiate into cells that express markers of the astrocyte, oligodendrocyte, and neuronal lineage when grown in serum-containing medium [42]. This culture forms invasive tumor when injected into the brains of immunocompromised mice [42] and was recently shown to be of the classical GBM subtype [42, 44]. T86 was maintained in serum-free neural stem cell medium as previously described [42] at 36°C in a standard tissue culture incubator (95% humidity, 95% air, and 5% CO2). To validate the expression of MMP-2 by GBM cells, T86 cells were seeded onto a poly-l-lysine coated (Sigma-Aldrich, Germany) 24-well plate (20000 cells/well) with 1 ml serum-free medium/well. After five days, the medium was discarded, and cells were fixed in 4% neutral-buffered formaldehyde for 10 min. Next, cells were washed three times in Dulbecco's phosphate-buffered saline (PBS, Gibco, USA), and primary antibodies against glial fibrillary acidic protein (GFAP, clone Z0334, Dako, Denmark, diluted at 1:1000), MMP-2 (diluted 1:100) or TIMP-1 (1:500) were added and allowed to incubate overnight. After washing in PBS, cells were incubated with secondary antibodies: donkey anti-Mouse IgG (heavy and light chain) Alexa Fluor 555 (MMP-2 and TIMP-1) or goat anti-Rabbit IgG (heavy and light chain) Alexa Fluor 488 (GFAP) (Thermo Fisher Scientific, USA) for 1h (diluted at 1:100). Cells were counterstained using Hoechst 33342 (Thermo Fisher Scientific). Images were recorded by a fluorescence microscopy (Leica DM IRB) and digital camera (Leica DFC 300 FX). The experiment was performed in duplicates. Omission of primary antibodies served as negative controls.

### Statistical analysis

Student’s unpaired t-test was used for comparison of two groups, while one-way analysis of variance (ANOVA) with Bonferroni correction was used when comparing more than two groups. Correlation analyses were performed using the non-parametric Spearman’s correlation. Kaplan-Meier survival curves were constructed and compared using the univariate log rank test. The median was used as a pre-specified cutoff value. The multivariate Cox regression model was used to adjust for age and gender or to adjust for GBM subtype, age and gender in the TCGA dataset. Overall survival was defined from the day of primary surgery until death or censoring (last evaluated February 12, 2016). All assumptions were tested, and survival analyses and multivariate Cox regression analyses were performed in STATA version 14 (StataCorp LP, USA). Other analyses were carried out in GraphPad Prism 5.0 (GraphPad Software Inc., USA). The statistical significance level was defined as p-value <0.05.

## Results

### MMP-2 staining pattern

**Fig 2:**
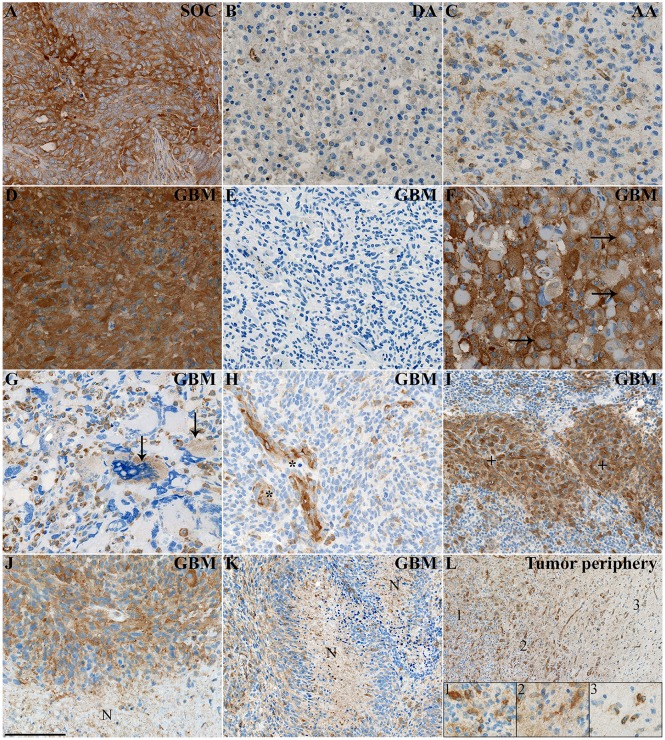
Immunohistochemical stainings of MMP-2. **(A)** MMP-2 showed intense expression in the serous ovarian carcinoma (SOC) which was used as a positive control. **(B-C)** MMP-2 was weakly expressed in diffuse astrocytomas (DA), while it exhibited stronger expression levels in anaplastic astrocytomas (AA). **(D)** The majority of glioblastomas (GBMs) had areas with intense MMP-2 expression. **(E)** However, a few GBMs were mostly MMP-2 negative. **(F)** Gemistocytic tumor cells often expressed high levels of MMP-2, especially in the membrane (*horizontal arrows*). **(G)** Multinucleated giant cells expressed limited amounts of MMP-2 (*vertical arrows*). **(H)** Blood vessels (*asterisks*) including **(I)** glomeruloid blood vessels (*plus signs*) were largely MMP-2 positive and often exhibited intense staining. **(J-K)** Cells surrounding larger necrotic areas (*N*) often showed intense MMP-2 positivity, while the intensity appeared lower in areas with pseudopalisading necroses (*N*). **(L)** MMP-2 positive cells were present in higher amounts in the central tumor (*insert 1*), but some intensely stained cells were also found in the border zone (*insert 2*) as well as in the tumor periphery (*insert 3*), where there was a considerably lower cellularity. Scale bar 100 μm (A-H, J), 400 μm (I, K, L).

Immunohistochemistry was performed on 89 astrocytic brain tumors. Patient characteristics are described in Table 1. For some patients, normal brain tissue was present in the biopsy material; in the normal tissue, only a few cells were positive for MMP-2, mainly with vascular/perivascular localization (Fig 1A). Serous ovarian carcinoma which was used as a positive staining control showed intense MMP-2 expression (Fig 2A).

**Table 1:** Patient characteristics and MMP-2 intensity.

Parameter	DA	AA	GBM	All astrocytomas
**Patients (n)**				
	11 (12%)	6 (7%)	72 (81%)	89 (100%)
**Gender (n)**				
Male	6 (55%)	3 (50%)	46 (64%)	55 (62%)
Female	5 (45%)	3 (50%)	26 (36%)	34 (38%)
**Age**				
Mean	51.7	60.4	61.3	60.3
Range	23.3–78.5	29.6–77.3	21.2–78.4	21.2–78.5
**Status (n)**				
Alive	1 (9%)	0 (0%)	1 (1%)	2 (2%)
Dead	10 (91%)	6 (100%)	71 (99%)	87 (98%)
**OS (months)**				
Median	56.3	18.4	8.4	8.9
Range	2.1–247.8	2.2–110.1	0.07–160.0	0.07–172.8
**MMP-2**				
Mean ± SD	101.4 ± 11.3	104.9 ± 12.8	118.5 ± 10.1	115.5 ± 12.1

**Abbreviations:** AA anaplastic astrocytoma; DA diffuse astrocytoma; GBM glioblastoma; MMP-2 matrix metalloproteinase-2; OS overall survival; SD standard deviation

In astrocytomas, MMP-2 was mainly expressed in the cytoplasm, but was also present in the membranes and ECM. Most DAs had low levels of MMP-2 (Fig 2B), while MMP-2 expression appeared more distinct and intense in AAs (Fig 2C). In general, GBMs expressed high levels of MMP-2 (Fig 2D), but a few GBMs exhibited very low MMP-2 intensities (Fig 2E). In GBMs, most gemistocytic tumor cells showed intense MMP-2 membrane expression (Fig 2F), whereas multinucleated giant cells generally were negative or only weakly expressed MMP-2 (Fig 2G). In all tumor grades, especially in GBMs, blood vessels were largely MMP-2 positive (Fig 2H). Similar was observed for microvascular proliferations such as glomeruloid tufts (Fig 2I). Perinecrotic areas were often densely surrounded by cells with intense MMP-2 expression (Fig 2J); while the density appeared lower in areas with pseudopalisading necroses (Fig 2K). In the border zone between central tumor and tumor periphery as well as in the tumor periphery, MMP-2 was diffusely expressed, but some tumor cells with intense MMP-2 expression were observed (Fig 2L).

To support that tumor cells express MMP-2, we performed immunofluorescence on a GBM cell culture (T86). We found that all GBM cells expressed the tumor marker GFAP, and some expressed MMP-2 and TIMP-1 both in their cytoplasm and membrane (S2 Fig).

### MMP-2 expression and tumor malignancy

**Fig 3:**
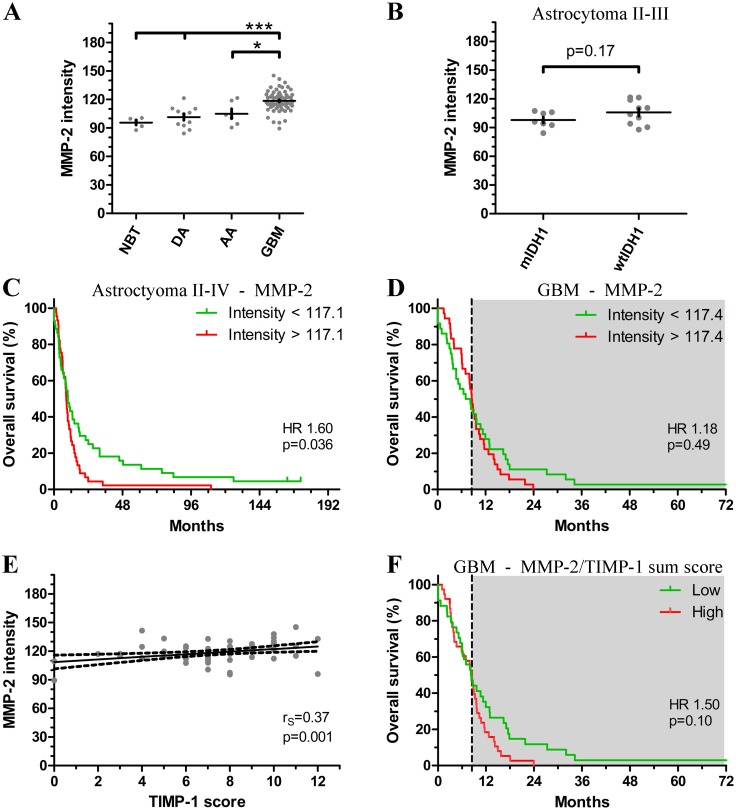
MMP-2 expression and tumor aggressiveness. **(A)** Scatterplot showing that MMP-2 intensity increases with tumor grade. **(B)** Patients with wildtype IDH1 (wtIDH1) astrocytomas tended to have higher MMP-2 intensity compared to patients with mutated IDH1 (mIDH1) astrocytomas. **(C)** High MMP-2 intensity informed poorer prognosis in patients with grade II-IV astrocytomas. **(D)** In GBMs, MMP-2 seemed to be a time-dependent variable (*dotted vertical line*). When accounting for the time-dependency, high levels of MMP-2 were associated with poorer prognosis in patients who lived longer than 8.5 months (*grey box*). MMP-2 had no prognostic value when the time-dependency was not accounted for. **(E)** MMP-2 intensity showed positive correlation with TIMP-1 score. **(F)** Combining TIMP-1 and MMP-2 expression, patients with high sum score tended have shorter overall survival when dichotomized at the median and without adjusting for time-dependency. Accounting for the time-dependency, high sum score was associated with shorter overall survival in patients living longer than 8.5 months (*dotted vertical line* and *grey box*). Abbreviations: AA anaplastic astrocytoma; NBT normal brain tissue; DA diffuse astrocytoma; GBM glioblastoma multiforme; mIDH1 mutant isocitrate dehydrogenase 1; wtIDH wildtype isocitrate dehydrogenase 1. * indicates p-value <0.05; *** indicates p-value < 0.001. *Vertical lines* indicate mean ± standard deviation.

The average MMP-2 expression increased with WHO tumor grade (Fig 3A and Table 1). GBMs had higher MMP-2 intensity compared to normal brain tissue (mean: 95.7 ±5.6; p<0.001), DAs (p<0.001) and AAs (p<0.05). Investigating the association between IDH1 status and MMP-2 levels in grade II-III astrocytomas, patients with wildtype IDH1 tumors (wtIDH1, n = 10; mean: 106.0 ±4.0) tended to have higher MMP-2 intensity than patients with mutated IDH1 tumors (mIDH1, n = 7; mean: 97.9 ±3.2) (p = 0.17) (Fig 3B). In patients with GBM, there was no significant difference in MMP-2 intensity between wtIDH1 (n = 69; mean: 118.6 ± 1.2) and mIDH1 tumors (n = 3; mean: 117.3 ± 3.6) (p = 0.84).

### MMP-2 expression and patient prognosis

High MMP-2 expression was significantly associated with poorer survival in patients with grade II-IV astrocytoma when dichotomized at the median intensity of 117.1 (HR 1.60; 95% CI 1.03–2.48; p = 0.036) (Fig 3C). Survival analyses were not performed for DA (n = 11) or AA (n = 6) due to the limited number of patients. In patients with GBM, median MMP-2 intensity was 117.4 (range: 89.3–145.2). When divided at the median, MMP-2 intensity was not prognostic (HR 1.18; 95% CI 0.73–1.92; p = 0.49) (Fig 3D). However, MMP-2 intensity seemed to be a time-dependent variable. When including the time-dependency in the multivariate analysis, MMP-2 intensity was not significantly associated with prognosis the first 8.5 months following diagnosis (HR 0.72; 95% CI 0.38–1.38; p = 0.32). After 8.5 months, patients with high levels of MMP-2 had a significantly poorer overall survival compared to patients with low intensity (HR 2.18; 95% CI 1.05–4.56; p = 0.036) (Fig 3D). This was also significant in the multivariate analysis when adjusting for age and gender (HR 2.27; 95% CI 1.07–4.81; p = 0.033) (Table 2).

**Table 2:** Multivariate analysis of MMP-2 expression.

	Patients (n)	HR (95% CI)	p-value
**Age (continuous)**			
	-	1.00 (0.99–1.04)	0.29
**Gender**			
Male	46	1.00	
Female	26	0.97 (0.58–1.61)	0.90
**MMP-2 intensity first 8.5 months**			
Low	20	1.00	
High	17	0.73 (0.38–1.40)	0.34
**MMP-2 intensity after 8.5 months**			
Low	16	1.00	
High	19	2.27 (1.07–4.81)	**0.033**

### Association between MMP-2 and TIMP-1 expression

To investigate the association between MMP-2 and TIMP-1 in GBMs, MMP-2 intensity was correlated with total TIMP-1 score. A significant positive correlation was found between the two proteins (r_S_ = 0.37; 95% CI 0.15–0.56; p = 0.001) (Fig 3E). As previously reported, we found low total TIMP-1 score to predict longer survival in patients with GBM (p = 0.001) [10]. When combing MMP-2 expression and TIMP-1 score into a sum score, high sum score tended to associate with poorer overall survival (HR 1.50; 95% CI 0.92–2.45; p = 0.10) when dividing at the median sum score of 4 (Fig 3F). The sum score appeared to be time-dependent; adjusting for the time-dependency, the sum score was prognostic after 8.5 months (HR 2.72; 95% CI 1.29–5.73; p = 0.009) (Fig 3F), while no prognostic impact was found in the first 8.5 months following diagnosis (HR 0.93; 95% CI 0.49–1.78; p = 0.83). When adjusting for age and gender, the sum score remained significant after 8.5 months (HR 2.78; 95% CI 1.30–5.92; p = 0.008) (Table 3).

**Table 3:** Multivariate analysis of sum score.

	Patients (n)	HR (95% CI)	p-value
**Age (continuous)**			
	-	1.01 (0.99–1.04)	0.29
**Gender**			
Male	46	1.00	
Female	26	0.96 (0.58–1.58)	0.86
**Sum score**^a^ **first 8.5 months**			
Low	18	1.00	
High	19	0.93 (0.49–1.78)	0.84
**Sum score**^a^ **after 8.5 months**			
Low	16	1.00	
High	19	2.78 (1.30–5.92)	**0.008**

^**a**^ the sum score is based on a summation of total TIMP-1 score and the MMP-2 group as patients with a MMP-2 intensity below the median intensity value (117.4) were given a score of 0, while patients with an intensity above 117.4 received a score of 1.

### mRNA expression of MMP-2 and TIMP-1 in TCGA

**Fig 4:**
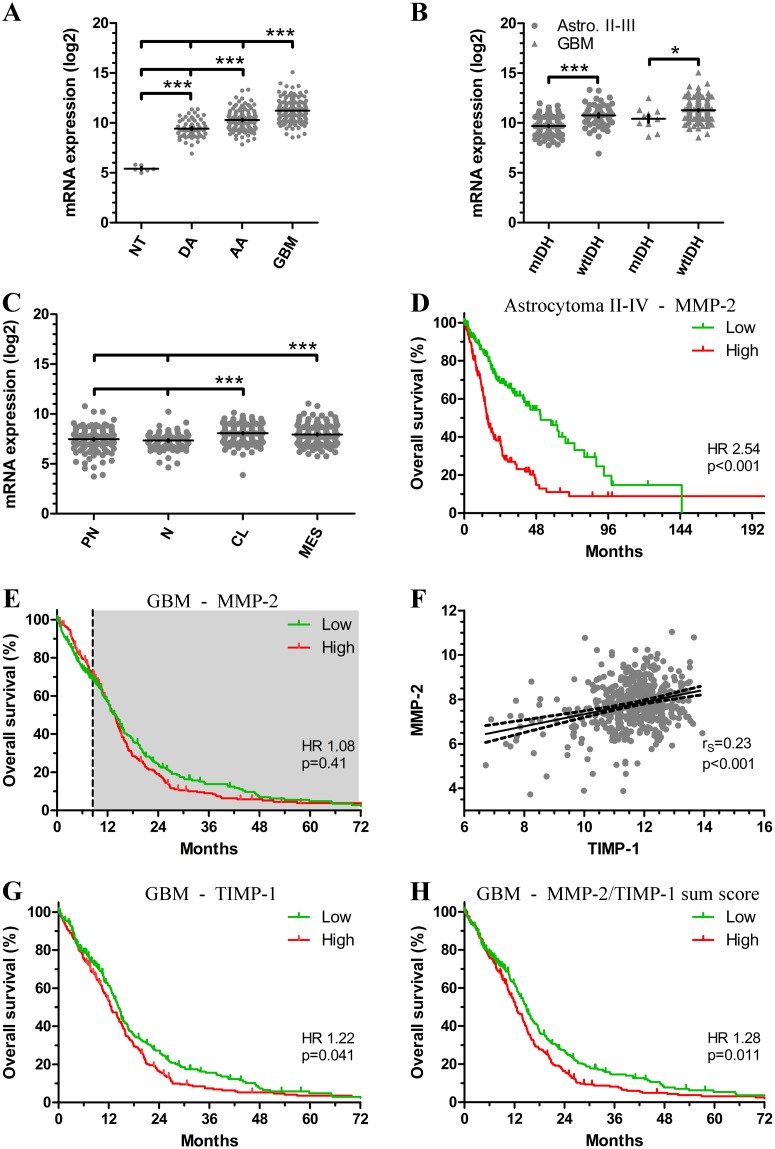
mRNA expression of MMP-2 and TIMP-1 in the TCGA. **(A)** The mRNA expression of MMP-2 increases with tumor grade. **(B)** Patients with wildtype IDH (wtIDH) tumors had significantly higher MMP-2 level than patients with mutated IDH (mIDH) tumors. **(C)** High MMP-2 expression was associated with shorter overall survival in grade II-IV astrocytomas. **(D)** In GBMs, MMP-2 was not associated with prognosis when dichotomized at the median and without accounting for the time-dependency. High levels tended to associate with poorer overall survival in patients who survived longer than 8.5 months (*dotted vertical line* and *grey box*). **(E)** MMP-2 expression was positively correlated with TIMP-1 expression. **(F)** In GBMs, patients with high TIMP-1 expression had poorer survival compared to patients with low levels. **(G)** Combining TIMP-1 and MMP-2 expression levels, patients with high sum score had poorer overall survival. Abbreviations: AA anaplastic astrocytoma; NT non-tumor tissue; DA diffuse astrocytoma; GBM glioblastoma multiforme; mIDH mutant isocitrate dehydrogenase; wtIDH wildtype isocitrate dehydrogenase. * indicates p-value <0.05; *** indicates p-value < 0.001. *Vertical lines* indicate mean ± standard deviation.

To further investigate the influence of MMP-2 and TIMP-1 on tumor aggressiveness and prognosis in patients with astrocytomas, we evaluated mRNA expression levels in TCGA. The mRNA expression of MMP-2 increased significantly with malignancy grade (p<0.001) (Fig 4A). In both grade II-III astrocytomas and GBM, patients with wtIDH tumors had higher mRNA levels than patients with mIDH tumors (p<0.001 and p = 0.017) (Fig 4B). When investigating the relation between GBM subtype and MMP-2, classical and mesenchymal GBMs had the highest MMP-2 levels compared to the proneural and neural GBMs (p<0.001) (Fig 4C). In survival analysis, high MMP-2 expression was associated with poorer outcome in patients with grade II-IV astrocytomas (HR 2.54; 95% CI 1.86–3.48; p<0.001) (Fig 4D). In patients with GBM, MMP-2 was not prognostic when dichotomized at the median (HR 1.08; 95% CI 0.89–1.31; p = 0.41) (Fig 4E), however, there seemed to be a time-dependency similar to that of the MMP-2 protein level. Adjusting for this, MMP-2 tended to associate with poorer patient survival after 8.5 months (HR 1.22; 95% CI 0.96–1.55; p = 0.099), while no association with prognosis was found in the first 8.5 months following diagnosis (HR 0.87; 95% CI 0.62–1.20; p = 0.39). Similar tendencies were found when adjusting for GBM subtype, age, and gender (Table 4). MGMT promoter methylation status did not significantly impact MMP-2 levels (p = 0.50) (S3 Fig) and was therefore not included in the multivariate analysis.

**Table 4:** Multivariate analysis of MMP-2 (TCGA).

	Patients (n)	HR (95% CI)	p-value
**Age (continuous)**			
	-	1.03 (1.02–1.04)	**<0.001**
**Gender**			
Male	307	1.00	
Female	189	0.93 (0.76–1.14)	0.46
**Subtype**			
PN	128	1.00	
N	79	1.03 (0.75–1.40)	0.85
MES	152	1.02 (0.78–1.32)	0.89
CL	138	0.90 (0.70–1.18)	0.43
**mRNA MMP-2 first 8.5 months**			
Low	149	1.00	
High	166	0.86 (0.62–1.20)	0.38
**mRNA MMP-2 after 8.5 months**			
Low	99	1.00	
High	83	1.19 (0.93–1.52)	0.16

Abbreviations: CL classical; MES mesenchymal; N neural; PN proneural

High MMP-2 expression was significantly correlated with high levels of TIMP-1 (r_S_ = 0.23, 95% CI 0.14–0.31; p<0.001) (Fig 4F). High TIMP-1 levels were associated with poorer survival in patients with GBM when divided at the median (HR 1.22; 95% CI 1.01–1.48; p = 0.041) (Fig 4G). When combining MMP-2 and TIMP-1 into a sum score, high levels were significantly associated with shorter overall survival (HR 1.28; 95% CI 1.05–1.55; p = 0.011) (Fig 4H), also when accounting for GBM subtype, age, and gender (HR 1.32; 95% CI 1.06–1.65; p = 0.015).

**Fig 5:**
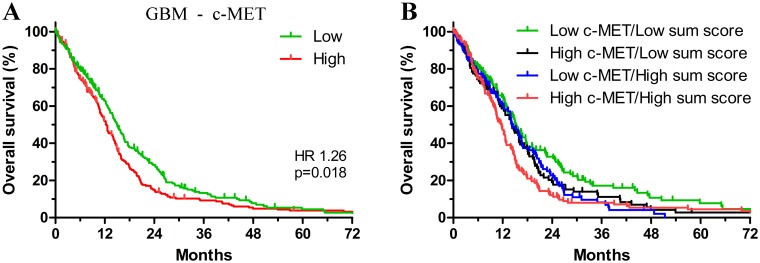
mRNA expression and impact of c-MET in the TCGA. **(A)** High levels of c-MET were significantly associated with reduced overall survival. **(B)** Stratifying patients into four groups based on c-MET and the sum score of MMP-2 and TIMP-1 showed that patients with high c-MET and high sum score had significantly poorer prognosis than patients with low c-MET and low sum score.

In a previous study we found that c-MET protein, a membrane receptor tyrosine kinase, similar to MMP-2 protein only informed shorter survival in GBM patients surviving longer than 8.5 months [30]. In the TCGA dataset, high c-MET mRNA was significantly associated with shorter survival (HR 1.26; 95% CI 1.04–1.52; p = 0.018), but c-MET mRNA did not appear to be a time-dependent prognosticator (Fig 5A). Stratifying TCGA patients into four groups based on c-MET and MMP-2/TIMP-1 sum score showed that patients with both high c-MET and high sum score had significantly reduced survival compared to patients with low c-MET and low sum score (HR 1.51; 95% CI 1.17–1.94; p = 0.001) (Fig 5B), and this was augmented when adjusting for GBM subtype, age, and gender in the multivariate analysis (HR 1.73; 95% CI 1.27–2.35; p<0.001) (Table 5).

**Table 5:** Multivariate analysis of c-MET and MMP-2/TIMP-1 sum score (TCGA).

	Patients (n)	HR (95% CI)	p-value
**Age (continuous)**			
	-	1.03 (1.02–1.04)	**<0.001**
**Gender**			
Male	307	1.00	
Female	189	0.95 (0.78–1.16)	0.62
**Subtype**			
PN	128	1.00	
N	79	0.96 (0.71–1.31)	0.82
MES	152	0.76 (0.56–1.04)	0.09
CL	138	0.78 (0.58–1.05)	0.10
**c-MET / sum score**			
Low/Low	144	1.00	
High/Low	104	1.26 (0.94–1.70)	0.12
Low/High	104	1.27 (0.93–1.72)	0.13
High/High	145	1.73 (1.27–2.35)	**<0.001**

Abbreviations: CL classical; MES mesenchymal; N neural; PN proneural

## Discussion

In the present study we investigated the immunohistochemical expression of MMP-2 in 89 astrocytic brain tumors and found that MMP-2 was expressed by tumor cells and blood vessels. The MMP-2 level increased with malignancy grade, and high expression was associated with shorter survival in patients diagnosed with grade II-IV astrocytomas as well as in patients with GBM who lived longer than 8.5 months after initial diagnosis. Combining the expression levels of MMP-2 and TIMP-1 seemed to be a stronger prognosticator of poorer outcome than MMP-2 alone.

In this study, we used quantitative digital image analysis which is a relatively objective quantitative approach compared to conventional scoring with intra- and inter-observer variation [45, 46]. When analyzing the expression of a biomarker with cytoplasmic or combined cytoplasmic/membrane localization, a classifier can be trained to grow a defined perimeter around all nuclei enabling measurement of staining intensity. Examples of combined cytoplasmic/membrane markers, which we have analyzed successfully using this approach, are junctional adhesion molecule-A (JAM-A), a tight junction molecule [29], c-MET [30], and now MMP-2. The final approval of a classifier is made by the observer and relies on whether the classifier is able to recognize and correctly classify e.g. tumor nuclei. The classifier will therefore never be better than the observer at recognizing nuclei, but the intra-observer variation is eliminated. The inter-observer variation is still a challenge in the automated quantification as the ultimate approval of the classifier is subjective. When using automated quantification, the overall goal should always be to design a classifier that accurately reflects the actual staining, and the training of the classifier should be performed by an observer blinded to information on malignancy grade and patient survival. Our classifier was trained by a group of observers to ensure robust measurements and circumvent/minimize inter-observer variation. To our knowledge, the present study is the first to utilize this method to investigate MMP-2 in astrocytic tumors.

In our study, MMP-2 was expressed by tumor cells in all tumor grades and was mainly located in the cytoplasm, but also in the membrane. Blood vessels were MMP-2 positive, also in normal brain parenchyma. This finding suggests that MMP-2 may be involved in angiogenesis as previously reported [26, 47, 48]. We further observed that perinecrotic areas often were surrounded by MMP-2 positive cells suggesting that hypoxia may induce expression of MMP-2 possibly resulting in neo-angiogenesis. This view is supported by results indicating that MMP-2 support cerebral vascular remodeling under hypoxic conditions [49].

In line with previous results [23–26, 50], we confirmed that MMP-2 is expressed in astrocytomas with the highest level in GBMs and found that high levels of MMP-2 were inversely related to survival in patients with grade II-IV astrocytic tumors. These results were validated in bioinformatics databases and indicate that MMP-2 is related to tumor biology and aggressiveness. To investigate the prognostic value of MMP-2, patients should by stratified into grades as grade itself predicts poorer survival. Due to small patient numbers, we could not perform survival analysis in grade II-III astrocytomas; instead we focused on the GBMs where the number of patients was sufficient for survival analysis. Jaalinoja et al. investigated the prognostic potential of MMP-2 in WHO grade II-III astrocytomas, but did not find a significant impact of MMP-2 on survival [23]. This could be due to low number of patients as 26 grade II-III astrocytomas were included. In pediatric gliomas, Gu et al. also found a significantly higher MMP-2 expression in high-grade compared to low-grade tumors [50]. In contrast to our findings, Kunishio et al. did not observe any association between tumor grade and MMP-2 expression or between prognosis and MMP-2 expression [51]. This discrepancy could be due to the use of different antibodies and immunohistochemical protocols, but also due to the limited number of patients in their study comprising 37 astrocytic tumors including 14 GBMs.

We further observed that patients with wtIDH1 tumors tended to have a higher MMP-2 expression than patients with mIDH1 tumors further supporting the idea that MMP-2 contributes to tumor aggressiveness as patients with mutations in IDH have a better prognosis, especially in WHO grade II-III [52]. This is supported by a recent report on lysyl oxidase (LOX) [53]. Besides being associated with tumor aggressiveness, LOX was lower in mIDH1 grade II and IV astrocytomas compared to wIDH1. Further, in vitro knockdown or inhibition of LOX in GBM cells resulted in diminished migratory and invasive abilities. Using chemotaxis assays, Wang et al. showed that U87 cells transfected with a mutated IDH1 gene had lower invasive capabilities, and this was linked to reduced levels of MMP-2 and MMP-9 [54]. In line with these results, Kessler et al. found that overexpressing the IDH1 mutation (IDH1^R132H^) in GBM cell lines decreased migration both under normoxic and hypoxic conditions [55]. Overall, this suggests that a mutation in IDH reduces the intratumoral production of pro-invasive proteins, possibly due to the metabolic/oxidative changes that follow from the mutation [56].

Recently, classification of GBM into subtypes has proven to have some value with regards to prognosis and response to treatment [57–59]. We therefore investigated the relation between MMP-2 and tumor subtype using the TCGA dataset. We found that MMP-2 levels were highest in mesenchymal and classical GBMs compared to proneural and neural GBMs. Classical GBMs are especially characterized by amplification in the epidermal growth factor receptor (EGFR), a receptor that has also been linked to tumor invasiveness [60]. Mesenchymal GBMs have been associated with poorer prognosis, treatment resistance, and higher degrees of necrosis [57, 61, 62], with the latter being an essential diagnostic criteria for GBM [63]. This GBM subtype is characterized by mutations in the tumor suppressors: phosphatase and tensin homolog (PTEN) and p53 [58, 59]. These molecular alterations are also frequent in giant cell GBMs and gliosarcomas [59]. Further, MMP-2 and MMP-9 were shown to be distinctly expressed in mesenchymal tumor areas of gliosarcomas [64]. Additionally, tumor-infiltrating lymphocytes were found to be enriched in mesenchymal GBMs [65] and in giant cell GBMs [59]. Interestingly, deletions in PTEN may be related to T cell anergy as well as tumor tolerance [66]. Summarized, these findings substantiate that MMP-2 contributes to tumor aggressiveness.

In GBMs, we found that MMP-2 was a time-dependent variable as high levels of MMP-2 were associated with poor survival only in patients who lived minimum 8.5 months. This was significant also when adjusting for age and gender. We found a similar tendency exploring the TCGA dataset when accounting for age, gender, and GBM subtype. We did not adjust for MGMT methylation status in the multivariate analysis as we found no association between MMP-2 levels and MGMT methylation status. Further, we did not account for IDH status as our patient cohort only contained three IDH1 mutated GBMs and seeing that IDH mutations are most prevalent in secondary GBM [67, 68]. The time-dependency of MMP-2 indicates that the expression levels of MMP-2 are only clinically relevant in patients who are alive at least 8.5 months following their primary diagnosis. One explanation could be that patients who die before 8.5 months have more advanced disease at the time of diagnosis thereby abolishing the prognostic value of proteins related to e.g. tumor invasion. Recently, c-MET was also shown to be time-dependent and influence prognosis in GBM patients who survived longer than 8.5 months [30], however, we were unable to validate this time-dependent prognostication in the TCGA dataset. In gliomas, c-MET and MMP-2 may share several properties promoting tumor survival, angiogenesis, and invasion [69, 70], and in fact similar to MMP-2, cells with strong c-MET expression were found in blood vessels and perinecrotic areas [30]. Further, the protein expression of hepatocyte growth factor (HGF), the ligand for c-MET, was found to positively correlate and be co-expressed with MMP-2 in human glioma [24].

We have previously reported that low TIMP-1 score predicts longer survival in patients with GBM [10]. We therefore wanted to investigate the association between TIMP-1 and MMP-2. We found a positive correlation between these two proteins, and the prognostic impact was stronger when we combined the expression levels of MMP-2 and TIMP-1 into a sum score compared to MMP-2 alone, overall suggesting that the two ECM-related proteins potentiate each other. These findings were confirmed in the TCGA dataset. Similar to both MMP-2 and c-MET, TIMP-1 positive cells were present around blood vessels as well as in areas of necrosis [10], and similar to MMP-2, cells expressing TIMP-1 were also observed in the tumor periphery. Using the TCGA dataset, we stratified patients into four groups based on c-MET and the sum score of MMP-2 and TIMP-1. We found that high levels of c-MET significantly worsened the prognosis for patients with high sum score. In summary, these results indicate that MMP-2 and TIMP-1 possibly aided by c-MET may promote tumor progression by enhancing angiogenesis and tumor invasion.

MMP inhibitors have been tested in patients with recurrent GBM, but without showing any significant improvements in survival [8]. In a phase II study cediranib, a pan-vascular endothelial growth factor receptor tyrosine kinase inhibitor, was tested in recurrent GBM patients, and a panel of circulating molecules including MMP-2 were evaluated in search of predictive biomarkers. Plasma MMP-2 levels were found to increase significantly during treatment and correlate with reduced progression-free and overall survival thereby being suggestive of poorer treatment response [71]. In contrast, in a study testing the efficacy of bevacizumab in patients with recurrent high-grade gliomas, high MMP-2 plasma levels were associated with improved tumor control and survival [72]. Overall, these studies suggest that MMP-2 may have some predictive value. Taking our results into account, treatment with MMP-2 and/or MMP-2/TIMP-1 inhibitors may primarily be efficient in patients who live longer than 8.5 months after initial diagnosis, but this should be investigating further in randomized clinical trials.

In conclusion, we demonstrated that MMP-2 expression is higher in GBMs compared to DAs and AAs. High levels of MMP-2 were associated with shorter survival in patients with grade II-IV tumors. In GBMs, MMP-2 was prognostic in patients who survived longer than 8.5 months, and this prognostic impact was intensified by TIMP-1. This suggests that MMP-2 contributes to tumor aggressiveness in astrocytic brain tumors and may have a prognostic potential in patients with GBM. However, the results need to be validated in a larger independent patient cohort.

## Supporting information

S1 Fig Impact of fixation time on MMP-2 intensity. Matrix metalloproteinase-2 (MMP-2) staining intensity was similar at different fixation times. **(A-D)** Tissue samples from the same glioblastoma patient fixated for 1h (**A**), for 6h (**B**), 12h (**C**), 24h (**D**) and 48h (**E**). Scale bar 100 μm.
(TIF)

S2 Fig Expression of GFAP, MMP-2 and TIMP-1 in a patient-derived glioblastoma cell culture (T86). T86 cells were seeded onto poly-l-lysine coated 24-well plates and grown in serum-free neural stem cell medium, resulting in growth of adherent cells of which some grew as spheroids. GFAP was expressed by all glioblastoma cells, but with diverse intensities (**A-D**). MMP-2 was expressed by some tumor cells at a medium to low intensity (**E-H**). Similar staining pattern was observed for TIMP-1 (**I-L**). Scale bar 100 μm.
(TIF)

S3 Fig Association between MGMT methylation status and MMP-2 levels in glioblastomas. Matrix metalloproteinase-2 (MMP-2) mRNA levels were not significantly influenced by MGMT promoter methylation status (p = 0.50).
(TIF)

S1 File Clinicopathological data on the 89 patients investigated in the current study. Data file containing clinical information on gender, age at diagnosis, survival status for each patient included in this study. The data file also contains quantitative data on MMP-2 levels, TIMP-1 score, sum score, and IDH1 mutation status.
(XLSX)

S2 File Datasets generated from the Cancer Genome Atlas (TCGA). File containing all data generated from the TCGA datasets and used in the current study.
(XLSX)

## Abbreviations

m-MGMT: methylated MGMT promoter
u-MGMT: unmethylated MGMT promoter
