# Supplementary figures and images for: Expression and prognostic impact of matrix metalloproteinase-2 (MMP-2) in astrocytomas
Source: PLoS One. 2017 Feb 24;12(2):e0172234. doi: 10.1371/journal.pone.0172234 (PMC5325257; doi:10.1371/journal.pone.0172234)

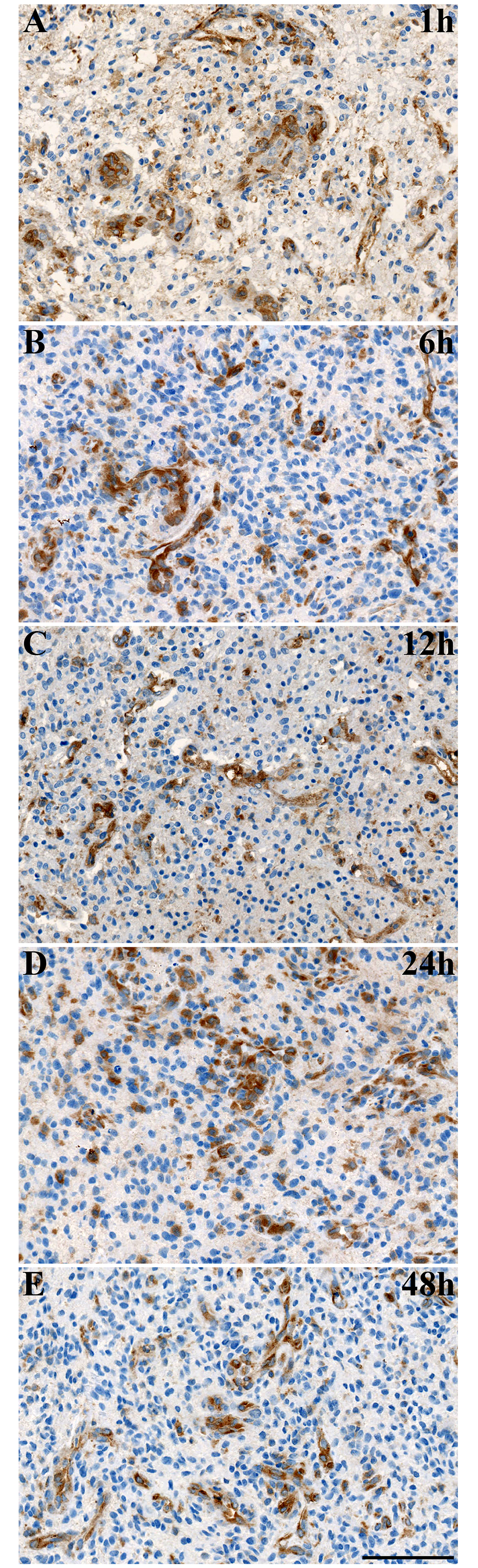

Supplement: S1 Fig — Matrix metalloproteinase-2 (MMP-2) staining intensity was similar at different fixation times. (A-D) Tissue samples from the same glioblastoma patient fixated for 1h (A), for 6h (B), 12h (C), 24h (D) and 48h (E). Scale bar 100 μm. (TIF) [file pone.0172234.s001.tif]

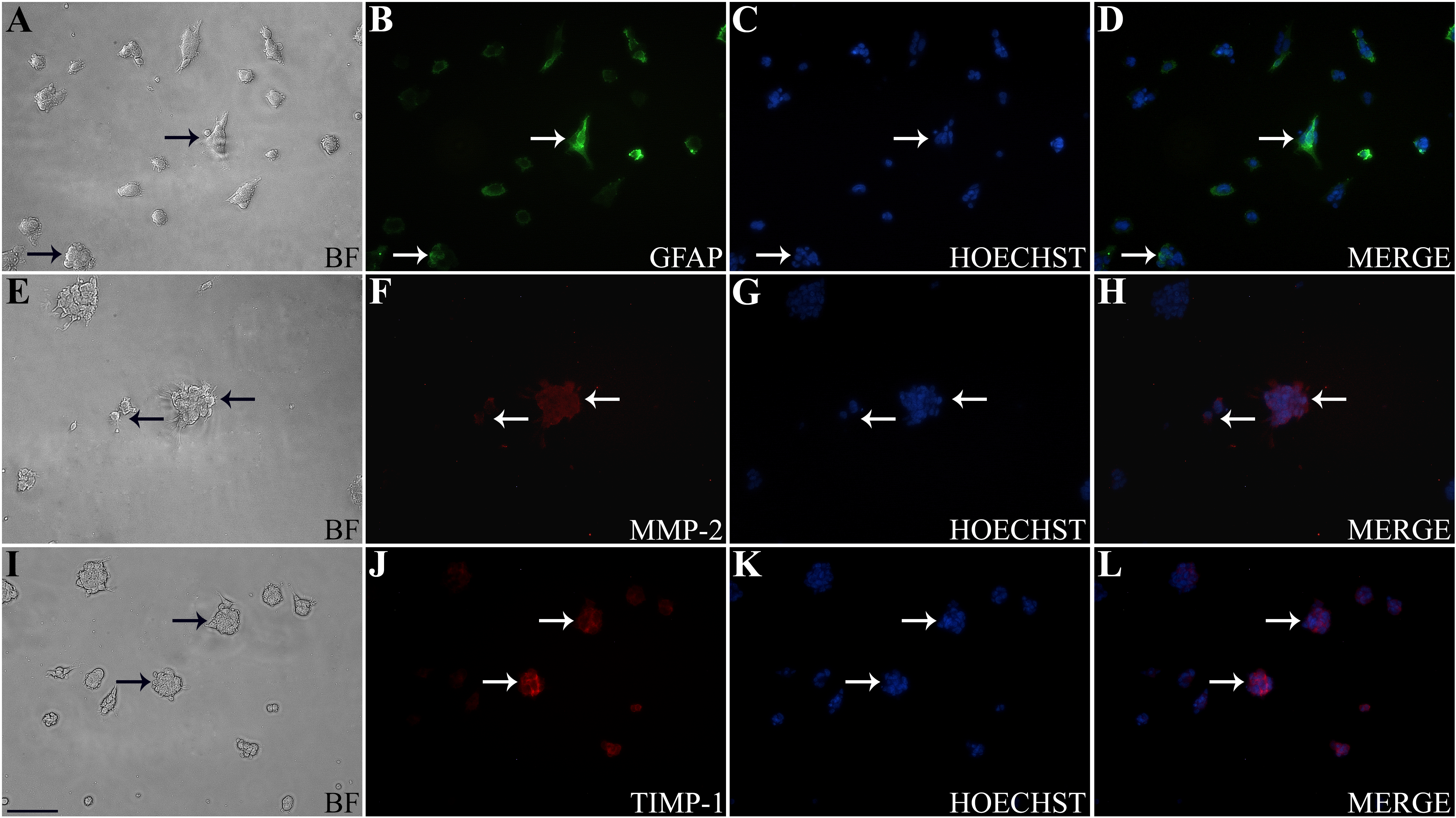

Supplement: S2 Fig — T86 cells were seeded onto poly-l-lysine coated 24-well plates and grown in serum-free neural stem cell medium, resulting in growth of adherent cells of which some grew as spheroids. GFAP was expressed by all glioblastoma cells, but with diverse intensities (A-D). MMP-2 was expressed by some tumor cells at a medium to low intensity (E-H). Similar staining pattern was observed for TIMP-1 (I-L). Scale bar 100 μm. (TIF) [file pone.0172234.s002.tif]

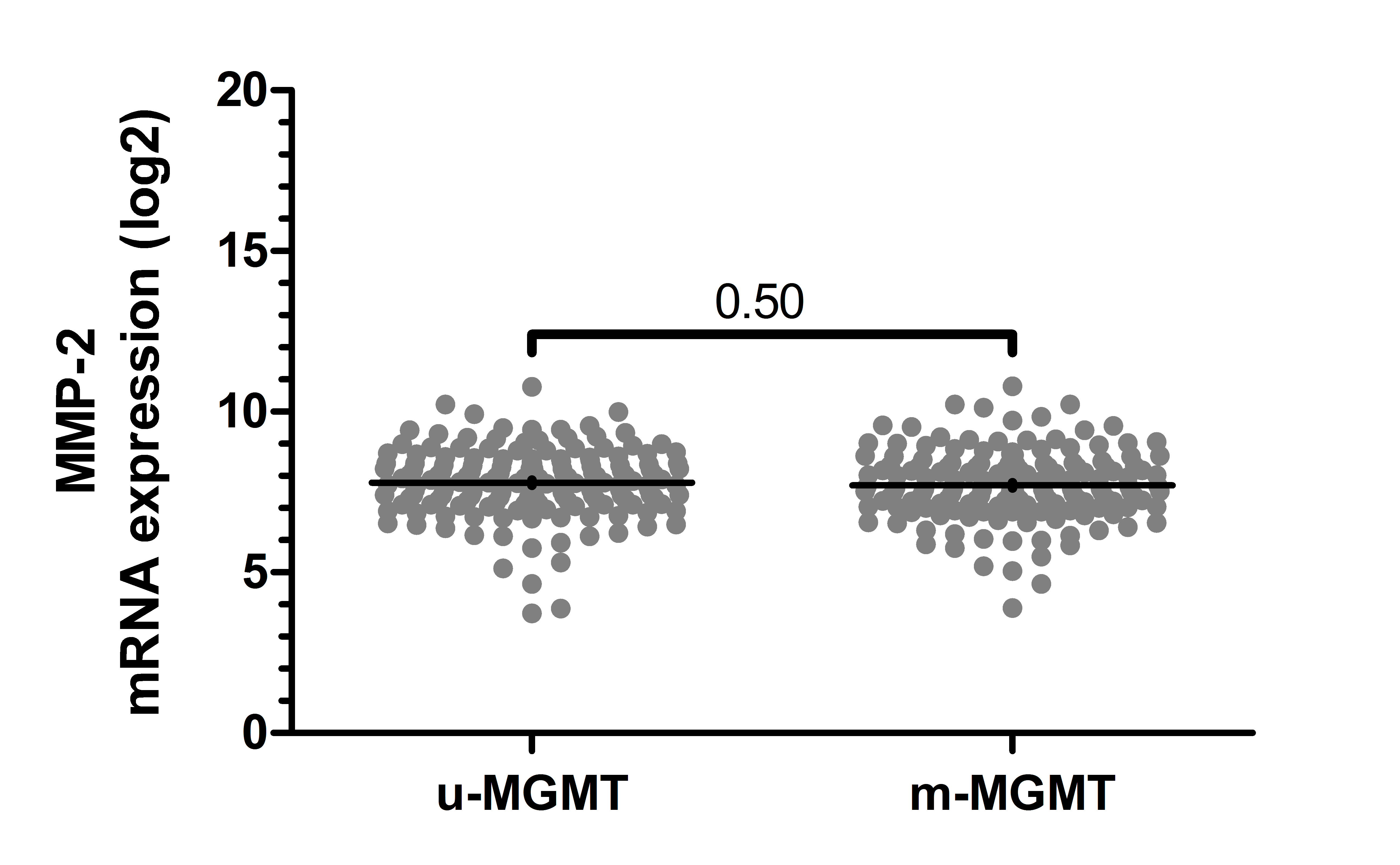

Supplement: S3 Fig — Matrix metalloproteinase-2 (MMP-2) mRNA levels were not significantly influenced by MGMT promoter methylation status (p = 0.50). (TIF) [file pone.0172234.s003.tif]
